# Structural Insights into the Role of Domain Flexibility in Human DNA Ligase IV

**DOI:** 10.1016/j.str.2012.04.012

**Published:** 2012-07-03

**Authors:** Takashi Ochi, Qian Wu, Dimitri Y. Chirgadze, J. Günter Grossmann, Victor M. Bolanos-Garcia, Tom L. Blundell

**Affiliations:** 1 Department of Biochemistry, University of Cambridge, 80 Tennis Court Road, Cambridge CB2 1GA, UK; 2 Molecular Biophysics Group, Institute of Integrative Biology, Faculty of Health and Life Sciences, Crown Street, The University of Liverpool, Liverpool L69 7ZB, UK

## Abstract

Knowledge of the architecture of DNA ligase IV (LigIV) and interactions with XRCC4 and XLF-Cernunnos is necessary for understanding its role in the ligation of double-strand breaks during nonhomologous end joining. Here we report the structure of a subdomain of the nucleotidyltrasferase domain of human LigIV and provide insights into the residues associated with LIG4 syndrome. We use this structural information together with the known structures of the BRCT/XRCC4 complex and those of LigIV orthologs to interpret small-angle X-ray scattering of LigIV in complex with XRCC4 and size exclusion chromatography of LigIV, XRCC4, and XLF-Cernunnos. Our results suggest that the flexibility of the catalytic region is limited in a manner that affects the formation of the LigIV/XRCC4/XLF-Cernunnos complex.

## Graphical abstract

**Graphical Abstract:**

**Figure:**
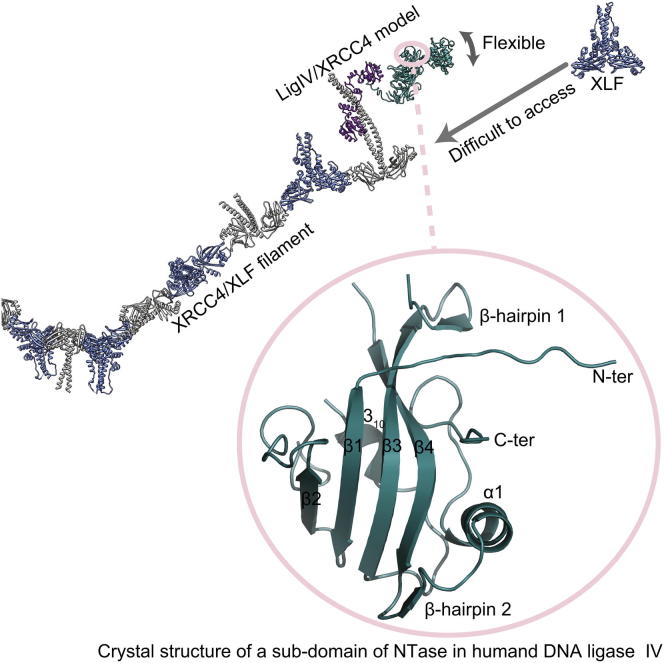

**Highlights:**

► Destabilization of the DNA ligase IV/XRCC4/XLF complex ► Structure of a subdomain of NTase of human DNA ligase IV at 2.9 Å resolution ► Flexibility and limited movement of the catalytic region of DNA ligase IV ► Structural insights into LIG4 syndrome

## Introduction

DNA ligase IV (LigIV) plays a major role in ligation of double-strand break (DSB) repair through nonhomologous end joining (NHEJ). This is carried out in three main steps. First, two DNA ends are brought together by DNA-dependent protein kinase (DNA-PK, a complex of the Ku70/80 heterodimer and the DNA-PK catalytic subunit [DNA-PKcs]) (Smith and Jackson, 1999). Second, the ends may be processed by nucleases and polymerases such as Artemis, polynucleotide kinase/phosphatase, and DNA polymerase γ and μ (Lieber, 2010). Finally, the ends are joined by ligase complex comprising LigIV, XRCC4, and XLF-Cernunnos (XLF) (Grawunder et al., 1997; Critchlow et al., 1997; Buck et al., 2006b; Ahnesorg et al., 2006).

DNA ligases I (LigI), III (LigIII), and LigIV share a common architecture of the catalytic region including the DNA-binding domain (DBD), the nucleotidyltransferase domain (NTase), and the OB-fold domain (OBD) (Ellenberger and Tomkinson, 2008). DBD was first identified in the structure of LigI and is important for DNA joining of the ligase (Pascal et al., 2004). The same domain was observed in LigIII (Cotner-Gohara et al., 2010) and archaeal DNA ligases (Pascal et al., 2006; Nishida et al., 2006; Kim et al., 2009). Six conserved motifs are present in NTase and OBD (Shuman and Schwer, 1995), which are important for the three steps of DNA ligation (Shuman and Lima, 2004). NTase has the ATP-grasp fold (Murzin, 1996) and is a member of the glutathione synthetase ATP-binding domain-like superfamily, comprising two subdomains that have the RAGNYA fold (Balaji and Aravind, 2007) and the phosphatidylinositol phosphate kinase (PIPK) C-terminal-like fold (Grishin, 1999). In the DNA-bound form of DNA ligases (Pascal et al., 2004; Nair et al., 2007; Cotner-Gohara et al., 2010), the subdomain with the RAGNYA fold is located at the 5′ end of a DNA nick (here called NTase-5), while the second subdomain lies at the 3′ end of a DNA nick (here called NTase-3).

In addition to these conserved catalytic domains, human DNA ligases have other domains at their N- and C-termini (Ellenberger and Tomkinson, 2008). LigIV has a tandem repeat of BRCT domains at its C-terminus, which is responsible for the interaction with its partner protein XRCC4 (Critchlow et al., 1997). Since LigIV is unstable without XRCC4 (Bryans et al., 1999), it is believed to be present predominantly in the complex form (LX4). Structural studies of human LX4 and its yeast ortholog Lig4p/Lif1p have shown that the BRCT domains of LigIV encircle the coiled-coil of the XRCC4 homodimer mainly via a conserved linker between the domains (Sibanda et al., 2001; Doré et al., 2006; Wu et al., 2009).

Negative-stain electron microscopy of the full-length complex (Recuero-Checa et al., 2009) indicate that the XRCC4 C-terminal domain (X4CTD) adopts a globular architecture giving rise to a compact structure of the whole complex. However, the failure of three-dimensional reconstruction of the complex implies heterogeneity in the conformation. SAXS (small angle X-ray scattering) studies of the BRCT domains and the full-length XRCC4 suggest that X4CTD is disordered but folds back toward the N-terminus of XRCC4 (Hammel et al., 2010). The structure of the catalytic region of LigIV and its relative arrangement with respect to the remaining part of LX4 remain unresolved.

LX4 forms a complex with XLF mainly via the head domains of XRCC4 and XLF (Ahnesorg et al., 2006; Deshpande and Wilson, 2007). Different groups have recently shown that the XRCC4/XLF complex assembles as a left-handed filament (Ropars et al., 2011; Hammel et al., 2011; Wu et al., 2011; Andres et al., 2012). Although XLF forms multimers in the presence of XRCC4 bound with the BRCT domains of LigIV (Andres et al., 2007; Hammel et al., 2010), the impact of the catalytic region of LigIV on XRCC4/XLF complex formation is unclear.

Here we report studies of LX4 using SAXS and X-ray crystallography to gain insights into the structure of the catalytic region of LigIV. We use gel filtration to demonstrate that the presence of the catalytic region destabilizes the LX4/XLF complex. A model of LigIV in complex with XRCC4 suggests that its limited flexibility leads to competition with the formation of the filament by XRCC4 and XLF.

## Results

### Interaction Studies of Human DNA Ligase IV/XRCC4 and XLF

**Figure 1:**
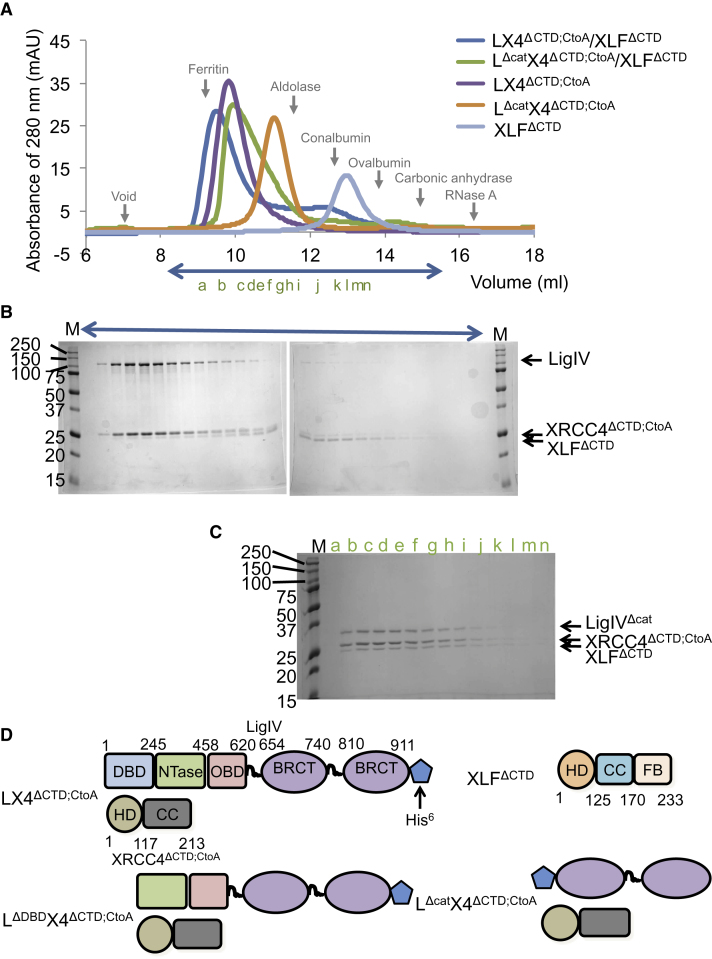
Gel Filtration Chromatography Studies of Complex Formation of LigIV, XRCC4^ΔCTD;CtoA^, and XLF^ΔCTD^ (A) Profiles of the UV absorbance at 280 nm during gel filtration chromatography. Colors of profiles and their corresponding constructs are shown at the bottom of the figure. Gray arrows indicate peak positions of protein standards, void, ferritin (440 kDa), aldolase (158 kDa), conalbumin (75 kDa), ovalbumin (43 kDa), carbonic anhydrase (29 kDa), and RNase A (13.7 kDa). (B) SDS-PAGE of LX4^ΔCTD;CtoA^ and XLF^ΔCTD^ fractions eluted from a Superdex 200 10/30 column. The molecular weight markers are in column “M” column and their molecular weights (kDa) are shown on the left of the gel. The fraction ranges used for the SDS-PAGE are indicated using a blue arrow in (A). Each fraction contained 250 μl of the eluted sample. (C) SDS-PAGE of L^Δcat^X4^ΔCTD;CtoA^ and XLF^ΔCTD^ eluted from the gel filtration column. The fractions used for SDS-PAGE are indicated alphabetically (green a-n) both in (A) and in the gel. (D) Schematic representation of the constructs used in the gel filtration experiment. The domain names and boundaries are shown in LX4^ΔCTD;CtoA^ and XLF^ΔCTD^. In XRCC4^ΔCTD;CtoA^ and XLF^ΔCTD^, HD, CC, and FB represent head, coiled-coil, and fold-back domains, respectively. See also Figure S1.

To define the effect of the catalytic region of LigIV on the XRCC4/XLF complex, we investigated the interactions between the three proteins using gel filtration chromatography. To eliminate the possibility of a contribution from this domain to the formation of the XRCC4/XLF complex and unnecessary higher order oligomer formation of XRCC4 though disulphide bonds, we used XRCC4^ΔCTD;CtoA^, where X4CTD (residues 214–334) is omitted and all cysteines are mutated to alanines. Interestingly, when the catalytic region of LigIV was present, LX4^ΔCTD;CtoA^ formed a less stable complex with human XLF lacking residues 234-299 (XLF^ΔCTD^) (Figures 1A and 1B); free XLF^ΔCTD^ was always observed in the constructs of the LX4 complex with the catalytic region. Similar results were observed with a full-length LX4 construct (data not shown). Only when XRCC4^ΔCTD;CtoA^ formed a complex with the BRCT domains alone (LigIV^Δcat^) could the two proteins interact stably with XLF^ΔCTD^ (Figures 1A and 1C). The removal of DBD from LigIV (LigIV^ΔDBD^) did not stabilize the complex between XRCC4^ΔCTD;CtoA^ and XLF^ΔCTD^ (Figures S1A and S1B available online). These results suggest that the catalytic core of LigIV prevents XLF^ΔCTD^ from binding the head domain of XRCC4^ΔCTD;CtoA^.

### SAXS Studies of Human DNA Ligase IV/XRCC4

**Figure 2:**
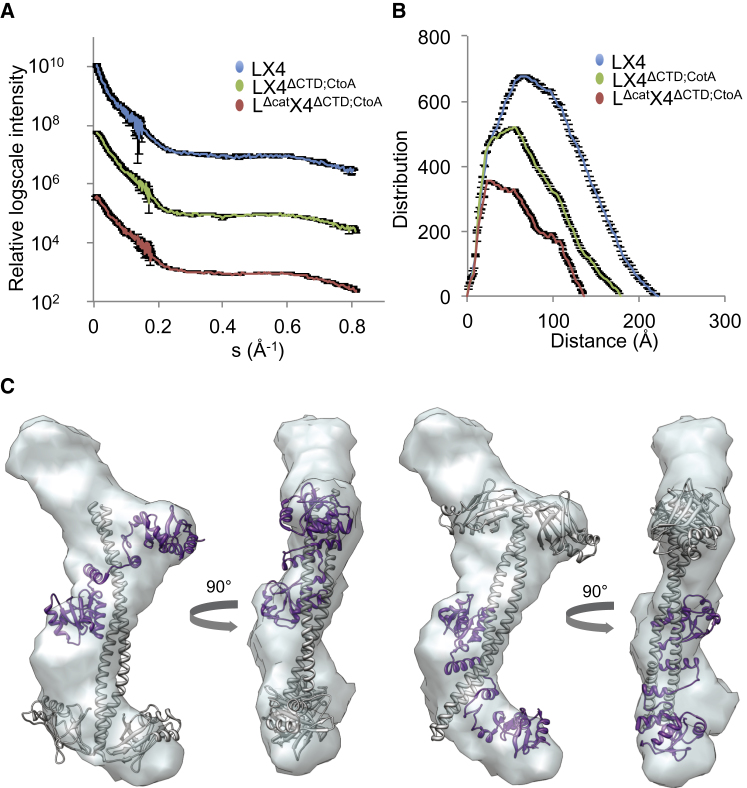
SAXS Studies of LX4 (A) Experimental scattering curves of LX4 constructs. The scattering intensities (log I versus s-value) with error bars (gray) of LX4 (blue), LX4^ΔCTD;CtoA^ (green) and L^Δcat^X4^ΔCTD;CtoA^ (blue) are displaced by factor of 100 for clarity. The scattering curves of the latter two constructs were modified after Ochi et al., 2010. (B) Distance distributions of LX4 constructs. The same color scheme as in (A) is used in this figure. The error bars are represented with gray. (C) Shape reconstruction of LX4^ΔCTD;CtoA^. The molecular envelope of LX4^ΔCTD;CtoA^ is shown in two perpendicular orientations, which derived from an averaging process of several, individually restored 3D shapes. The structure of a LX4 construct (PDB code: 3II6; Wu et al., 2009). The structure was fitted into the envelope manually and refined using Chimera (Pettersen et al., 2004). The two structural superimpositions providing the highest correlation correlations coefficients are illustrated to highlight additional molecular density not present in the crystal structure. See also Figure S2.

Because the gel filtration studies of the LigIV/XRCC4/XLF complex implied that the catalytic region of LigIV may have a well-defined conformation in relation to the remaining part of LX4, SAXS studies of the complex were carried out to investigate its overall shape. In addition to the full-length construct LX4, two other truncated constructs LX4^ΔCTD;CtoA^ and L^Δcat^X4^ΔCTD;CtoA^, the complex of LigIV^Δcat^ and XRCC4^ΔCTD;CtoA^ were investigated. The linearity of the Guinier plots [graph of the natural logarithm of the scattered intensity I(s) versus s^2^ at very low angles] suggested that the solution samples were well behaved and monodisperse (Figure S2A). *R*_g_ of LX4, LX4^ΔCTD;CtoA^ and L^Δcat^X4^ΔCTD;CtoA^ are 70.1, 55.3, and 46.3 Å, and their *D*_max_ are 222, 179, and 136 Å (Ochi et al., 2010). The deletion of X4CTD reduced *R*_g_ and *D*_max_ by 15 Å and 43 Å respectively, while deletion of the catalytic region further decreased these values by 9 Å and 43 Å, respectively. The scattering profiles of the three constructs have few prominent features, such as shoulders or inflection points, and these are primarily in the very low angle scattering region, thus indicating an inherent conformational plasticity (Figure 2A and 2B).

Ab initio 3D-shape reconstruction of LX4 did not provide strong evidence for a conserved molecular shape because ten individually restored models yielded conformations with a high degree of variation (emphasized by a NSD value of 1.06 for the average model). In contrast, reconstructions for LX4^ΔCTD;CtoA^ yielded NSD values of 0.81 for the average 3D shape (Ochi et al., 2010), which is elongated with an additional region of scattering density when the structure of L^Δcat^X4^ΔCTD^ (Wu et al., 2009), was fitted into the averaged envelope (Figure 2C; Figure S2C). The fitting was performed with eight different orientations, correlation coefficients (CCs) of which were calculated using UCSF Chimera (Pettersen et al., 2004) (models 1–8 in Figure S2D). Models 1 and 3 had the two highest CCs and extra densities were concentrated either near the BRCT domains of LigIV or the head domains of XRCC4 (left and right Figure 2C, respectively). These results imply that LX4 has an extended shape and the catalytic region of LigIV may have a well-defined structure near the BRCT or head domains.

### Crystallographic Structure of NTase-3 of Human DNA Ligase IV

**Figure 3:**
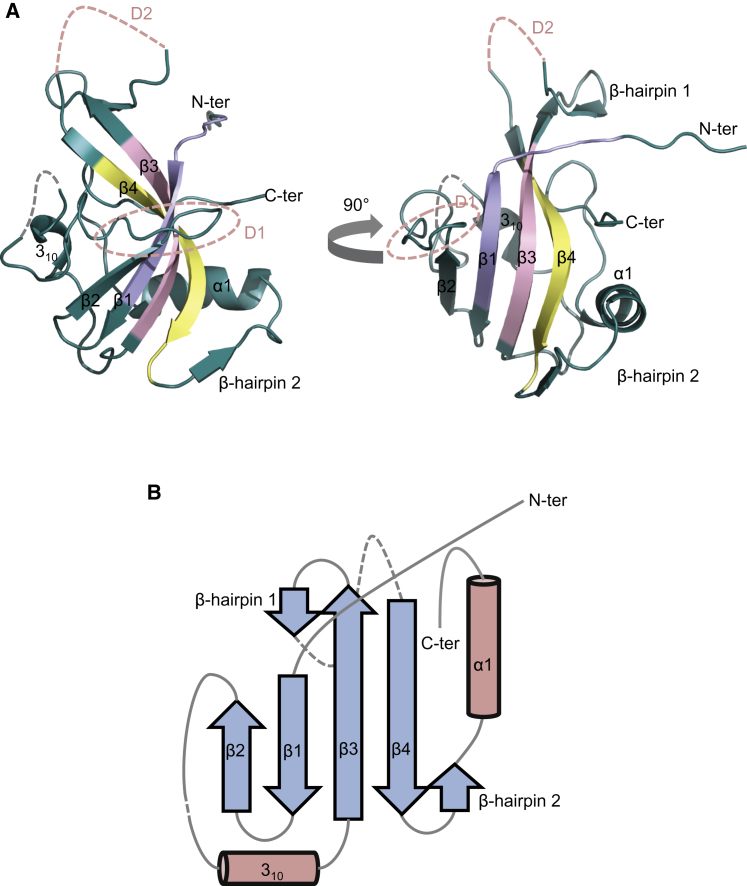
Structure of NTase-3 (A) Overall architecture of NTase-3. Conserved motifs I, III and IIIa are shown in purple, pink and yellow. Dotted lines represent missing loops (gray and pink) connecting DNA-binding regions D1 and D2 (pink). (B) A schematic presentation of secondary structure elements of NTase-3. See also Figure S3.

Although we gained information about the overall shape of LX4 from SAXS studies, the structural details of the N-terminal catalytic domains—DBD, NTase, and OBD—of LigIV remained unresolved. Therefore, we set out to define their structures by X-ray protein crystallography. We crystallized a subdomain of NTase (NTase-3) of human LigIV and solved the structure at the resolution of 2.9 Å using SAD and SIRAS methods (see Experimental Procedures for the details). The combination of the phases thus obtained provided electron density that allowed us to build a model of NTase-3 at 3.5 Å resolution. The model was further refined at 2.9 Å resolution, which gave an 84% complete model with an *R*/*R*_free_ of 27/30% (Table 1). The positions of methionine residues were confirmed by calculating the anomalous difference maps using the model and SeMet data (Figure S3B and Table 2). The structure revealed an overall architecture of the NTase-3 of LigIV that is similar to that of LigI (Pascal et al., 2004) and LigIII (Cotner-Gohara et al., 2010) (Figure S3C). LigIV has a six amino acid insert, which is relatively acidic, between β2 and 3_10_ defined by residues of low conservation across species (Figure 3A and Figure S4). The relative arrangement of motifs I, III, and IIIa resembles that of the other human DNA ligases, suggesting that LigIV carries out DNA ligation in a conventional manner. An extended polypeptide at the N-terminus (Figure 3B), which is supposed to form a β sheet in NTase-5 (Figure S5B), is stabilized by forming a fireman's grip with a corresponding peptide belonging to another asymmetric unit in crystal (data not shown).

### Rigid-Body Modeling of Human DNA Ligase IV/XRCC4

**Figure 4:**
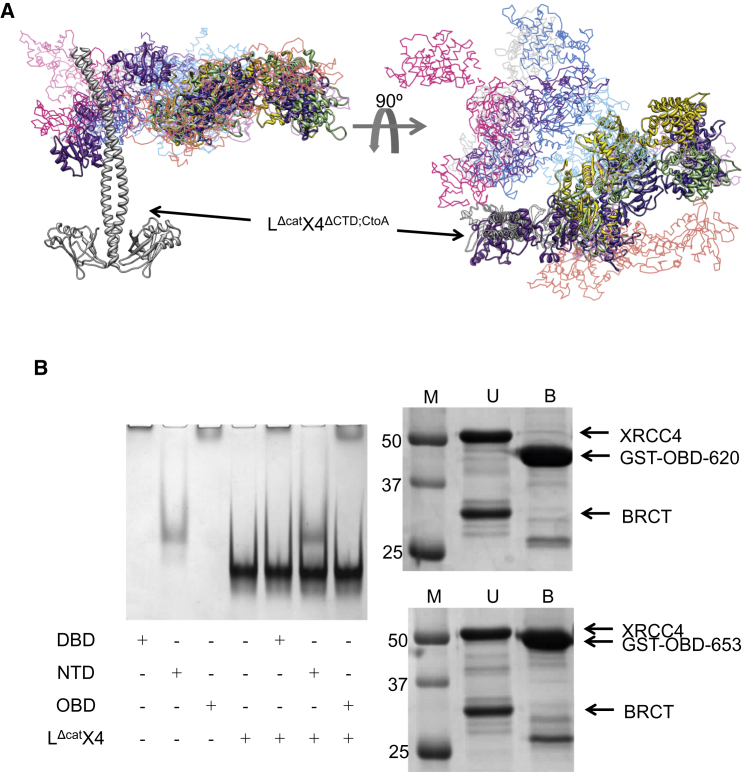
Rigid-Body Modeling and Protein-Protein Binding Assays of Human DNA Ligase IV/XRCC4 Complex (A) Rigid-body modeling of LX4^ΔCTD;CtoA^ using BUNCH. Ten individual rigid-body models were superposed on the structure of the L^Δcat^X4^ΔCTD;CtoA^ region. The models with the three highest χ^2^ values are shown in a cartoon representation and the others are shown as their Cα traces. (B) Left: EMSAs of individual catalytic domains and L^Δcat^X4. The proteins used are indicated with “+.” Right: GST pull-down assays of OBD and L^Δcat^X4. The upper and lower figures show the results of the assays using GST-OBD-620 and GST-OBD-653, respectively. The first lane protein markers (M) are followed by unbound proteins (U) and bound proteins (B) to GST affinity resin.

To gain further insights into the structure of the catalytic region of LigIV, homology models of DBD, NTase, and OBD were created using Modeller (Sali and Blundell, 1993). Then, the structure of NTase-3 was used as a template for the NTase model (see Experimental Procedures). The SAXS data of LX4^ΔCTD;CtoA^ were further analyzed by rigid-body modeling using BUNCH (Petoukhov and Svergun, 2005) as described in Experimental Procedures. The average χ^2^ of ten individual rigid-body models against the scattering data was 5.64 ± 0.50. Although they had differing conformations, the catalytic domains were found near the BRCT domain and were placed near the first BRCT domain in the models having the three lowest χ^2^ (Figure 4A). Taken together with the ab initio modeling of LX4^ΔCTD;CtoA^, our SAXS studies suggest that in solution the catalytic region stays near the BRCT domains rather than the head domain of XRCC4.

In order to investigate possible interactions between other structural units of the complex, we expressed and purified individual domains of the catalytic region: DBD, NTase, and OBD. Gel retardation assays of these domains, the BRCT domains and the XRCC4 (residues 1–334; L^Δcat^X4) domain did not demonstrate any interactions between them (Figure 4B, left). Since the OBD-620 (residues 457–620) used for the assays did not contain the linker region (residues 621–653), a further construct, OBD-653 (residues 457–653) was expressed with an N-terminal GST tag and purified. However, as this resulted in an insoluble protein after tag cleavage, OBD-653 was purified without removing the tag (GST-OBD-653). GST pull-down assays were carried out using GST-OBD-620 and GST-OBD-653. The results showed that GST-OBD-653 did not have a strong interaction with L^Δcat^X4 (Figure 4B, right). Thus, we were not able to substantiate an interaction between the catalytic region of LigIV and its BRCT domains and/or XRCC4.

### Insights into a DNA-Binding Region of NTase-3 of Human DNA Ligase IV

**Figure 5:**
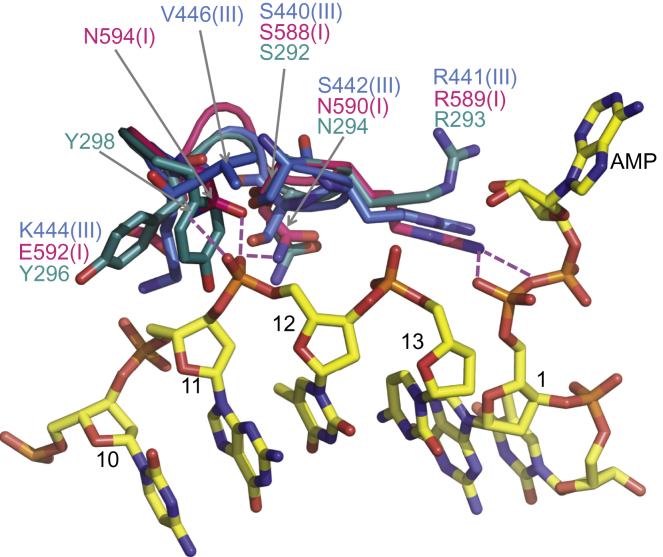
Comparison of the DNA-Binding Loop D1 of NTase-3 The structure of NTase-3 of LigIV (cyan) is shown together with that of LigI (I, pink; PDB code: 1X9N) and LigIII (III, blue; PDB code: 3L2P), and DNA (PDB code: 1X9N). Backbone phosphates of DNA are labeled as 12 and 13. The pink-dotted lines represent hydrogen bonds between LigI and the DNA. The original residue numbers of the phosphates shown in the PDB file are used here. See also Figure S4.

NTase-3 of DNA ligases has a DNA-binding region and loop immediately after β1 (D1) and the other between β3 and β4 (Figure S4). The latter is mostly disordered in the crystals studied here. The Ser/Arg motif in D1 (S292/R293), conserved in human DNA ligases (Figure S4), is located in a similar position to those in LigI and LigIII [S588(I)/R589(I) and S440 (III)/R441(III) in Figure 5]. In LigIV, position 298 is occupied by a tyrosine instead of the aspartate [D594(I)], which forms a hydrogen bond with the phosphate at position 12 of the DNA backbone in the LigI structure (Figure 5). At an equivalent position, LigIII has a conserved valine [V446(III) in Figure 5], which does not establish direct contacts with DNA but is involved in hydrophobic interactions to fix the DNA-binding region D1. With the exception of a few organisms in which histidines and phenylalanines are located in the equivalent position (data not shown), Y298 is conserved among LigIVs suggesting that this residue may play a role in DNA binding.

### LIG4 Syndrome Mutation in NTase-3 of Human DNA Ligase IV

**Figure 6:**
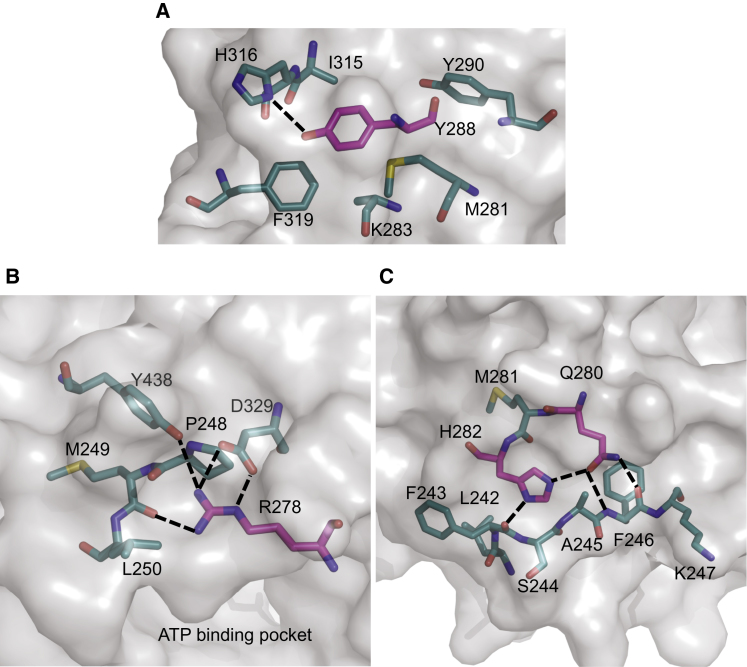
Interactions of Residues that Cause LIG4 Syndrome that Are Close to the Catalytic Residues of Human LigIV (A) Hydrophobic core of NTase-3 of LigIV around Y288 (magenta). Since the electron density for the side chains of K283 and I315 was not observed, the amino acids were represented as alanines. (B) Interactions in the model between R278 (magenta) and surrounding residues. (C) Interactions between Q280/H282 and surrounding residues. Salt bridges and hydrogen bonds are represented using black dotted lines. See also Figure S5.

R278, Q280, and H282, mutations of which cause LIG4 syndrome, are located on β1 in the same way as the equivalent residues of LigI and LigIII (Figure S5A). Because they probably play similar roles in DNA ligation, their mutation will likely lead to disruption of important interactions, as described below in the Discussion. A further mutation Y288C in mouse LigIV results in LIG4 syndrome (Nijnik et al., 2007). Some organisms have phenylalanine instead of tyrosine at this position (Figure S4). This tyrosine residue forms a part of the hydrophobic core of the NTase-3 domain and a hydrogen bond with H316 (Figure 6A), suggesting that the mouse Y288C substitution impairs the stability of NTase-3. As shown below, an analysis of the Y288C mutation indicates that it stabilizes the structure of NTase. However, the domain may not have an identical conformation, and this may affect the shape of the ATP-binding pocket and the activity of the enzyme.

To gain further insights into the role(s) of these residues, the structure of the entire NTase of LigIV was built using Modeller with the NTase-3 structure of LigIV and the experimentally defined domains from LigI (Protein Data Bank [PDB] code: 1X9N), LigIII (PDB code: 3L2P), and archeal DNA ligases (PDB codes: 2HIV, 2CFM, and 3GDE) as templates (Figure S5B). The possible effects of several substitutions were investigated using the SDM server (Worth et al., 2011). Solvent accessibilities of R278, Q280, H282 and Y288 were 15.9, 1.6, 0.1, and 0.1% respectively. Calculated pseudo ΔΔG values of R278H, Q280R, H282L, and Y288C substitutions found in LIG4 syndrome were 0.53, −1.50, 2.88, and 1.68, respectively. The mutations except for Q280R are predicted to stabilize the NTase domain. With the exception of R278, these residues are solvent inaccessible and highly hydrogen-bonded, suggesting that they play structural roles in NTase.

## Discussion

The overall shape of LX4 has been studied using SAXS in combination with X-ray crystallography and homology modeling. The position of the maximum of the distance distribution function is shifted toward the origin compared with *D*_max_/2, suggesting an elongated shape (Svergun and Koch, 2003). The Kratky plots of LX4^ΔCTD;CtoA^ and LX4 show characteristic bell-shaped profiles reminiscent of folded and/or compact macromolecules (Figure S2B). A SAXS study of *Sulfolobus solfataricus* DNA ligase, which contains the catalytic region only, indicates an open conformation with a *D*_max_ of about 120 Å in solution (Pascal et al., 2006). A similar result has been recently reported for the catalytic region of LigIII; however, an advanced SAXS data analysis strategy has revealed multiple conformations of LigIII in solution, although 74% of them are open structures (Cotner-Gohara et al., 2010). These reports imply that LigIV is likely to adopt a predominantly open conformation, a notion that is supported by our rigid-body analysis LX4^ΔCTD;CtoA^ from SAXS data. Because *D*_max_ of L^Δcat^ X4^ΔCTD;CtoA^ is 136 Å, the observed *D*_max_ of LX4^ΔCTD;CtoA^ should be over 200 Å instead of 179 Å if the catalytic region of LigIV had an extended structure. LX4 has an additional 43 Å extension, which is a contribution from X4CTD. If the domain were folded back toward the head domain of XRCC4 giving a compact structure, it should not increase the size of LX4^ΔCTD;CtoA^ to that extent. Thus, X4CTD may be flexibly linked with the other domains. This is supported by a SAXS study of the BRCT domains of LigIV and full-length XRCC4 (Hammel et al., 2010).

The SAXS study in solution of LX4^ΔCTD;CtoA^ defines an ensemble of extended and compact conformations, and rigid-body modeling indicates that in some of these the catalytic region is likely to be located near the BRCT domains. Indeed, the catalytic region of LigIV may be flexibly connected to the domains but limited in its movement, as suggested by electron microscopy studies of negative stained LX4 (Recuero-Checa et al., 2009). It has also been suggested that the flexibility of the catalytic region of LigIV could be required to facilitate the repair of various types of DSBs (Perry et al., 2010).

The limited movement of the catalytic region might be partly responsible for the inefficiency of the re-adenylation of LX4. It is known that DNA ligases with DBD, NTase, and OBD domains undergo large conformational changes in order to adenylate NTase (Pascal et al., 2004). Thus, if the movement of the catalytic region is restricted, the efficiency of re-adenylation will be reduced. However, the initial adenylation of LigIV is highly efficient: about 99% of LX4 was pre-adenylated in cells (Chen et al., 2009). Since LigIV in the free form is also likely to be difficult to re-adenylate (Wang et al., 2007), the highly efficient initial adenylation of LigIV seems to be a unique feature of this protein. The interaction with XRCC4 likely causes a conformational change in LigIV, which stimulates LigIV adenylation.

Our gel filtration data show that the presence of the catalytic region of LigIV destabilizes the formation of the XRCC4/XLF complex. Although we observed the formation of the LigIV/XRCC4/XLF complex, it was less stable than the complex using L^Δcat^X4^ΔCTD;CtoA^ (Figures 1B and 1C). Since we also observed the L^Δcat^X4^ΔCTD;CtoA^/XLF complex using gel filtration (Figures 1A and 1C), EMSA (data not shown), and electrospray ionization mass spectrometry (data not shown), our results are still compatible with results reported by others showing that L^Δcat^X4^ΔCTD^ in the presence of XLF forms filaments (Andres et al., 2007; Hammel et al., 2010). The XRCC4/XLF filament is likely to exist given that there are more XRCC4 molecules than LigIV in vivo (Mani et al., 2010). However, the structure of the filament may be altered in the presence of the full-length LigIV. The removal of DBD from LigIV did not greatly affect the destabilization of the XRCC4/XLF complex, suggesting that NTase and/or OBD may clash with XLF. These results imply that the catalytic core is located, for at least some of the time, where it can cause stereochemical clashes with XLF, probably near the head domain of XRCC4. Alternatively, because XLF seems to have physical contacts with the BRCT1 (Wu et al., 2009), it may compete with the catalytic region of LigIV. Ku70/80 might also mediate the interaction because it was detected by pull-down assays using whole cell extracts of MRC5 cells, and both XLF, which is DNA-dependent (Yano et al., 2008), and BRCT1 (Costantini et al., 2007) interact with Ku70/80. It is reported that XLF and its yeast ortholog Nej1 assist re-adenylation and de-adenylation of LigIV (Riballo et al., 2009; Chen and Tomkinson, 2011). XLF might free the catalytic region to make it possible to change its conformation for re-adenylation. If the catalytic region of LigIV is located near BRCT1, one of the head domains of XRCC4 in the LX4 is more likely to interact with XLF than the other; thus, LX4 may prefer to occupy a position at the end of the XRCC4/XLF filament. This would ensure that the ligase interacts with Ku70/80 and DNA ends for DSB end joining.

The structure of NTase-3 and a homology model of NTase of LigIV demonstrate the structural importance of R278, Q280, H282 and Y288. The homology model of NTase of LigIV indicates that R278 will likely establish a hydrogen bond with the carbonyl oxygen of M249 and a salt bridge with D329 (Figure 6B). Interestingly, substitution of M249 to valine also results in LIG4 syndrome (Toita et al., 2007). Since these residues belong to the other subdomain of NTase (NTase-5; see Introduction), the interactions may stabilize the structure of the catalytic pocket by fixing the relative position of NTase-3 and -5. In addition, in the structure of LigI, the corresponding arginine makes a hydrogen bond with the 3′-OH of the AMP ribose (Pascal et al., 2004). The corresponding arginine (R39) makes the same hydrogen bond with ATP in T7 DNA ligase (Subramanya et al., 1996). Indeed, the mutation to histidine impairs the interaction with ATP resulting in inefficiency of adenylation as observed experimentally (Riballo et al., 1999). Therefore, the substitution R278H is likely to lead to instability of the ATP-binding pocket and the interaction with ATP. A similar proposal has been made on the basis of the structure of T4 DNA ligase (Riballo et al., 2001).

The catalytic pocket is further stabilized by Q280 and H282 via interactions with the N-terminal peptide of NTase (residue 242–249) (Figure 6C). LIG4 syndrome mutant Q280R may fail to form hydrogen bonds with the peptide, and, as a result, might destabilize the catalytic pocket of NTase. V(D)J recombination was undetectable in *LIG4* gene null cells that instead carry the *LIG4^Q280R^* gene; however, in vitro studies of the Q280R mutant showed that it joined DNA nicks as efficiently as the wild-type protein (Buck et al., 2006a). This implies that the Q280R mutant has normal ligation activities. Since there are hydrophobic residues around H282 (Figure 6C), the mutation H282L is likely to result in the stabilization of NTase as suggested by the SDM analysis. However, the mutation no longer makes the hydrogen bond with Q280 and may change the conformation of the catalytic pocket. Because experimental data of the H282L mutant are unavailable, it is difficult to assess the impact of the mutation. Since R278, Q280, and H282 all interact with the N-terminal peptide of NTase, it is likely that the peptide is important for stabilizing the conformation of the catalytic pocket of NTase. This is also supported by a recent report demonstrating that conserved buried polar residues that are hydrogen-bonded are important for the stabilization of protein structures (Worth and Blundell, 2010). Interestingly, the peptide and the following β strand are unique to polynucleotide ligase and mRNA capping enzymes in the glutathione synthetase ATP-binding domain-like superfamily. Thus, they may be important for polynucleotide binding. Apart from R278 they are not directly involved in the catalytic activity of LigIV, so the instability of the ATP-binding pocket is likely to impair the LigIV/AMP complex formation (Riballo et al., 1999; O'Driscoll et al., 2001). Another LIG4 syndrome mutant R814X, which lacks the second BRCT domain, does not form the LigIV/AMP complex (Girard et al., 2004), suggesting that the adenylation of LigIV is very inefficient but not null in the mutations causing LIG4 syndrome. This means that we should be able to rescue the adenylation of the LigIV mutants if we could develop drugs that are analogs of ATP, which bind the catalytic pockets of the proteins and form a lysine/AMP intermediate.

In summary, we have described experiments that shed further light on both the structure of individual domains and the ensemble of conformers adopted by DNA ligase IV. X-ray analysis of NTase-3 of human LigIV has defined the structure of this subdomain and suggested structural roles for the residues, mutations of which cause LIG4 syndrome. With respect to the complete structure of this multimodular enzyme, we have shown that there is only limited movement of the catalytic region of DNA ligase IV and this prevents formation of a continuous filament formation of the XRCC4/XLF complex. Crystallographic studies of the LigIV/XRCC4/XLF complex, including the catalytic region, remain of central importance to gain insights into the regulation of complex formation and the nature of interactions at DSB sites.

## Experimental Procedures

### Purification of Human LigIV/XRCC4 Constructs

The LX4 co-expression plasmid was a gift from Prof. Ming-Daw Tsai. LX4 was expressed in Rossetta2(DE3) (Invitrogen) and purified as described previously (Wang et al., 2007). The *XRCC4* gene was amplified from an XRCC4^ΔCTD;CtoA^ expression plasmid, the protein of which has a residue range between 1 and 213 only and all cysteines are mutated to alanines (Sibanda et al., 2001) and cloned between NdeI and AvrII sites of the multiple cloning region (MCR) 2 of pRSFDuet1 vector (Novagen). Then, the *LIG4* and *LIG4^ΔDBD^*, which do not have DBD, were amplified from the co-expression plasmid and cloned between NcoI and EcoRI sites of MCR 1 of the pRSFDuet1. LX4^ΔCTD;CtoA^ and L^Δcat^X4^ΔCTD;CtoA^ were expressed in Rossetta2(DE3)pLysS (Invitrogen). Genes of individual domains of LigIV, DBD (residues 1–244) and OBD (458–620) were amplified from the LX4 co-expression plasmid and cloned into pGAT3 vectors (J. Peränen and M. Hyvönen, personal communication), which carry GST tags at their N-termini. NTase (residue 245–457) was cloned into pHAT5, which has a hexa-histidine tag at the C-terminus (Peränen et al., 1996). The domains were expressed in BL21(DE3) (Invitrogen). Purification protocols of these constructs are described in Supplemental Experimental Procedures.

### Size Exclusion Chromatography

1800 pmol of XLF^ΔCTD^ was mixed with the same quantity of LX4^ΔCTD;CtoA^, L^Δcat^X4^ΔCTD;CtoA^ or L^ΔDBD^ X4^ΔCTD;CtoA^ and incubated in 50 μl of reaction buffer (20 mM Tris-HCl pH 8.0 at 4°C, 150 mM NaCl, 5% [v/v] glycerol, 5 mM DTT) on ice for 60 min before loading onto a Superdex 200 10/300 (Pharmacia) column equilibrated with reaction buffer; 1800 pmol of those proteins were also individually loaded onto the column without incubation. High- and low-molecular weight markers (GE Healthcare) were loaded onto the column by following their guidelines.

### Crystallization and Data Collection

Ten mg/ml of NTase was mixed with 1/100 volume of 10 mg/ml papain from JBS Floppy-Choppy (Jena Bioscience) just before the crystallization trials of NTase. In the hanging drop method used here, 1.5 μl of crystallization agents (6%–8.5% [w/v] PEG6000, 100 mM MES pH 5.5–5.7, 4% [v/v] 2-propanol) were added to each drop containing an equal volume of the mixed protein solution on a siliconized cover glass placed against reservoir containing 600 μl of the agents. SeMetNTase was crystallized in a similar way in a different crystallization solution (17% [w/v] PEG6000, 100 mM MES pH 6.2, 10 mM betain hydrochloride). The sequence of the crystallized protein was confirmed by SDS-PAGE (Figure S3A) followed by mass spectrometry and N-terminal sequencing conducted by Dr. Len Packman and Mr. Mike Weldon at the PNAC Facility in the Department of Biochemistry, Cambridge University. To prepare a heavy atom derivative of the NTase, crystals were soaked in their reservoir solutions containing 1 mM thiomersal (Hg) for 2 hours or 0.1 mM osmium chloride (Os) overnight. Crystals of NTase and SeMetNTase were transferred to cryo-protectant solutions (30% of ethylene glycol mixed with 70% of crystallization agents) and frozen in liquid nitrogen. X-ray diffraction data collection experiments were carried out at either ESRF (Grenoble, France) or Diamond (Oxford, UK) synchrotron radiation sources. One native dataset (native-2) was collected using an in-house rotating anode X-ray generator (Proteum X8, Bruker AXS) at the X-ray crystallographic facility at the Department of Biochemistry, University of Cambridge to obtain a high redundancy dataset.

### Data Processing

Crystallographic data statistics of the collected X-ray diffraction datasets of NTase-3 are described in Table 2. X-ray diffraction data of the derivative crystals were processed using *MOSFLM* (Battye et al., 2011). Native-1 dataset was processed using HKL suite (Otwinowski and Minor, 1997), whereas native-2 dataset was processed using PROTEUM2 (Bruker AXS, Madison, WI). The scaling of the integrated data was carried out using *Scala* (Evans, 2006) for the derivative data and Scalepack (Otwinowski and Minor, 1997) for the native datasets. The space group of the crystals was determined as P4_1_22 based on the reflection conditions of the crystal, a self-rotation function calculated from Molrep (Vagin and Teplyakov, 1997) and the results of Pointless (Evans, 2006).

### Structure Solution and Model Building

Experimental phases were calculated from (1) the SeMet data using SAD methods, (2) Os and Hg data sets (Os/Hg phases) using SIRAS methods, and (3) native-2 and Hg data sets (native-2/Hg phases) using SIRAS methods. Multi-crystal averaging of these data was carried out using DMMULTI (Cowtan, 1994) to calculate phases for native-1 at the maximum resolution of 3.5 Å. The model of NTase-3 was built using Coot (Emsley et al., 2010). The phenix.refine and phenix.autobuilt modules in PHENIX suite with secondary structure and Ramachandran restraints (Adams et al., 2010) and Refmac 5.5 were used for refinement of the model. The refinement was then carried out using native-1 up to 2.9 Å resolution using PHENIX and the jelly-body refinement in Refmac 5.6 (Murshudov et al., 2011). The refinement and re-building of the model using Coot were repeated until there was no further improvement; the *R*/*R_free_* of the final model were 27/30%, respectively (Table 1). Each molecule representation was generated with PyMOL (Delano Scientific, San Carlos, CA) or UCSF Chimera (Pettersen et al., 2004). A more detailed description of the model building can be found in the Supplemental Experimental Procedures.

### Modeling of the Catalytic Region of Human DNA Ligase IV

Sequences of orthologs of LigIV were obtained using protein BLAST (Altschul et al., 1990) and aligned using Muscle (Edgar, 2004). For the sequence alignment of LigIVs, 11 nonredundant sequences were selected. In parallel, protein structures of LigIV homologs (human LigI [PDB code: 1X9N]; Pascal et al., 2004), human LigIII (PDB code: 3L2P; Cotner-Gohara et al., 2010), and three archeal DNA ligases (PDB codes 2HIV [Pascal et al., 2006], 2CFM [Nishida et al., 2006], and 3GDE [Kim et al., 2009]) were downloaded from RSBC PDB (Berman et al., 2000), and were structurally aligned using BATON (D.F. Burke, personal communication). The structural alignment template files were converted to FUGUE input files, and then aligned with the Muscle alignment files using Fugueali (Shi et al., 2001). The combined alignment files were represented in JOY format (Mizuguchi et al., 1998) and manually refined using SeaView (Gouy et al., 2010) when necessary.

Structural models of the individual catalytic domains of LigIV were created using Modeller (Sali and Blundell, 1993) based on the sequence alignment of the DNA ligases. As for the model of NTase, the crystallographic structure of NTase-3 of LigIV was included in the structural templates described above. The best model that had the lowest object function was selected from 30 created models. The predicted impact of amino acid residue substitutions of NTase was carried out with the program SDM (Worth et al., 2011).

### Small-Angle X-Ray Scattering

SAXS data collection was performed at station 2.1, Synchrotron Radiation Source, Daresbury Laboratory UK, with a two-dimensional multiple-wire proportional counter. The radius of gyration, the maximum particle dimension and the distance distribution function were calculated with GNOM (Svergun, 1992). DAMMIN (Svergun, 1999) was used for the ab initio shape reconstruction of the proteins. Subsequent rigid-body modeling of LX4^ΔCTD;CtoA^ was performed using BUNCH (Petoukhov and Svergun, 2005), after which we used the crystallographic structure of the complex of the BRCT domains of LigIV and XRCC4 (PDB code: 3II6; Wu et al., 2009), and homology models of DBD, NTase, and OBD (see above). The UCSF Chimera package (Pettersen et al., 2004) was used to visualize the model envelopes. See Supplemental Experimental Procedures for the details.

### Electrophoretic Mobility Shift Assay for Protein-Protein Interactions

Protein-protein interaction studies using electrophoretic mobility shift assays were carried out based on a published protocol (Andres et al., 2007). Proteins were incubated in 20 μl of the binding buffer (20 mM Tris-HCl pH 7.5 at 25°C, 50 mM KCl, 0.1 mM DTT, 5% [v/v] glycerol) at 25°C for 60 min. The incubated samples were directly loaded into Tris-HCl pH 8.0 at 4°C 5% polyacrylamide gel and separated by electrophoresis at 80 V in TBE for 110 min. The gel was stained and visualized with Coomassie blue.

### GST-Pull-down Assays

GST-pull-down assays were carried out according to a reported protocol (Einarson, 2001). Ten micrograms of GST fusion proteins were mixed with 1:1 molar ratio of L^Δcat^X4, which is the complex of the BRCT domains of LigIV (residues 654–911) with a N-terminal hexa-histidine tag and XRCC4 (residues 1–334). They were incubated with 25 μg GST resin at 4°C in the reaction buffer (20 mM Tris-HCl pH 8.0 at 4°C, 200 mM NaCl, 1 mM EDTA, 0.5% [v/v] NP-40) for 120 min. After washing the resin twice with the reaction buffer, the bound proteins were eluted with the reaction buffer plus 20 mM reduced glutathione.

## Figures and Tables

**Table 1:** Values for the Structural Model of NTase-3

Resolution (Å)	30.6–2.9
*R*_cryst_^a^ (%) (the highest shell)	27.3 (30.4)
*R*_free_^b^ (%) (the highest shell)	30.5 (39.1)
Structural model	
Number of atoms (non H)	952
RMSD bond (Å)	0.014
RMSD angle (°)	1.971
Ramachandran outliers^c^ (%)	0.8
Rotamer outliers^c^ (%)	7.4

a *R*_cryst_ = Σ‖*F*_obs_|-|*F*_calc_‖/Σ|*F*_obs_|, *F*_obs_ and *F*_calc_ are observed and calculated structure factor amplitudes.

b *R*_free_ as for *R*_cryst_ using a random subset of the data (about 10%) excluded from the refinement.

c Calculated with phenix.refine module of PHENIX suite (Adams et al., 2010).

**Table 2:** X-Ray Diffraction Data

Crystal	Native-1	Native-2	SeMet	Hg	Os
Source Beamline	ESRF ID14-1	In-house PROTEUM X8	ESRF ID14-1	ESRF ID14-1	Diamond I02
Wavelength (Å)	0.9765	1.5418	0.9795	1.007	1.140
Resolution (Å)	100-2.90	33.48-3.50	65.02-3.50	65.19-3.40	97.54-3.60
Space group	P4_1_22	P4_1_22	P4_1_22	P4_1_22	P4_1_22
Cell (Å)					
a = b	39.09	38.93	39.00	39.14	39.22
c	197.39	196.98	195.07	195.56	195.37
Number of unique reflections	3860	2278	2223	2447	2127
Completeness (%) (the highest resolution shell)	98.8 (100)^a^	99.4 (98.3)	99.9 (100)	99.9 (99.9)	100 (100)
Redundancy	11.9	72.2	12.0	12.1	11.9
*R*_sym_^b^ (%) (the highest shell)	6.3 (54.4)	33.98 (107)	9.4 (44.6)	9.4 (67.0)	13.0 (63.8)
*I*/σ (the highest shell)	15.3 (5.2)	18.3 (0.9)	14.8 (5.2)	14.6 (4.2)	10.3 (4.1)

a The numbers in parentheses represent the statistics for the highest resolution shell.

b *R*_sym_ = ∑_h_|*I*_h_ - < *I* > |/∑_h_*I*_h_, where *I*_h_ is the intensity of reflection h, and < *I* > is the mean intensity of all symmetry-related reflections.

## Accession Numbers

The PDB accession number for the coordinate of NTase-3 of human DNA ligase IV, together with the structure factors, reported in this paper is 3VNN.

## Supplemental Information

Document S1. Figures S1–S5 and Supplemental Experimental Procedures
