# Supplementary material for: Structural Insights into the Role of Domain Flexibility in Human DNA Ligase IV
Source: Structure. 2012 Jul 3;20-20(7):1212–22. doi: 10.1016/j.str.2012.04.012 (PMC3391681; doi:10.1016/j.str.2012.04.012)
Supplement: Document S1. Figures S1–S5 and Supplemental Experimental Procedures [file mmc1.pdf]

Structure, Volume *20*

Supplemental Information

Structural Insights into the Role of Domain Flexibility in Human DNA Ligase IV

Takashi Ochi, Qian Wu, Dimitri Y. Chirgadze, J. Günter Grossmann, Victor M. Bolanos-Garcia, and Tom L. Blundell

Inventory of Supplemental Information

Suppl. Figure 1. Additional gel filtration results related to Figure 1.

Suppl. Figure 2. Guinier and Kratky plots and additional 3D reconstructions related to Figure 2.

Suppl. Figure 3. SDS-PAGE of dissolved NTase crystals, anomalous difference maps and comparison of NTase-3 among human DNA ligases related to Figure 3.

Suppl. Figure 4. Sequence alignment of NTase-3 related to Figure 5.

Suppl. Figure 5. Conserved residues, mutations of which in human DNA ligase IV cause LIG4 syndrome related to Figure 6.

Supplemental Experimental Procedures

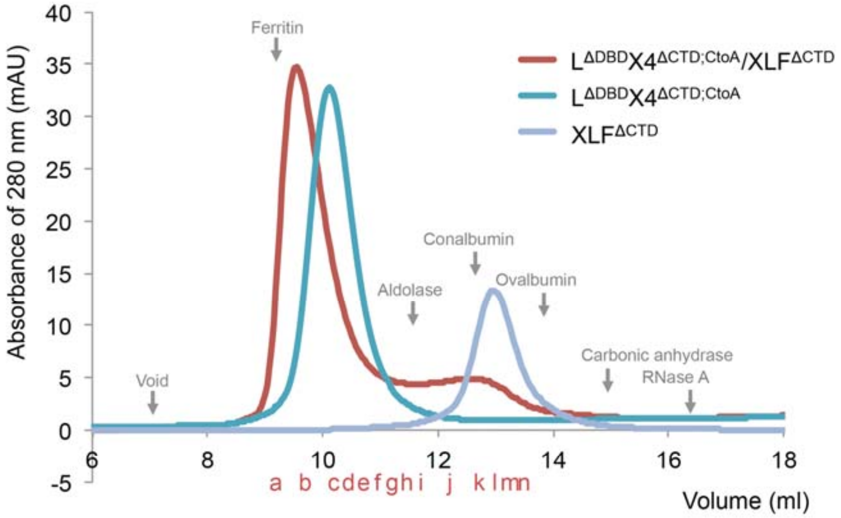

A

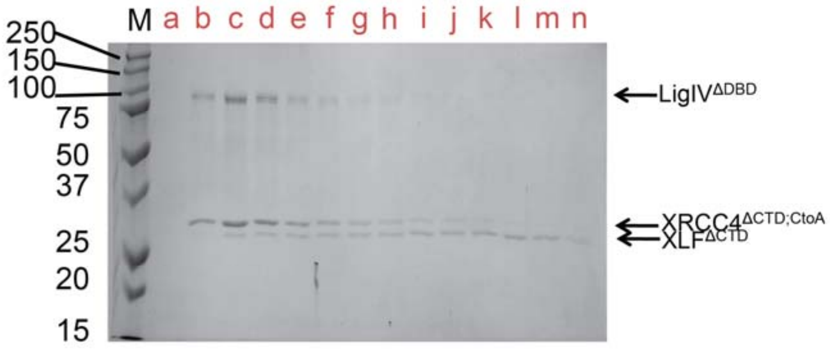

B

Figure S1, related to Figure 1. Gel filtration studies of complexes formed by LigIV$^{\Delta DBD}$, XRCC4$^{\Delta CTD;CtoA}$ and XLF$^{\Delta CTD}$. (A) Profiles of the UV absorbance at 280 nm during gel filtration. Colours of profiles and their corresponding constructs are shown at the upper left of the figure. Grey arrows indicate peak positions of void volume and protein standards: ferritin (440 kDa), aldolase (158 kDa), conalbumin (75 kDa), ovalbumin (44 kDa), carbonic anhydrase (29 kDa) and RNase A (13.7 kDa). (B) SDS-PAGE of L$^{\Delta DBD}$X4$^{\Delta CTD;CtoA}$ and XLF$^{\Delta CTD}$. The molecular weight markers are shown in the first lane. The fractions used for SDS-PAGE are indicated alphabetically (pink a-n) both in (A) and the gel.

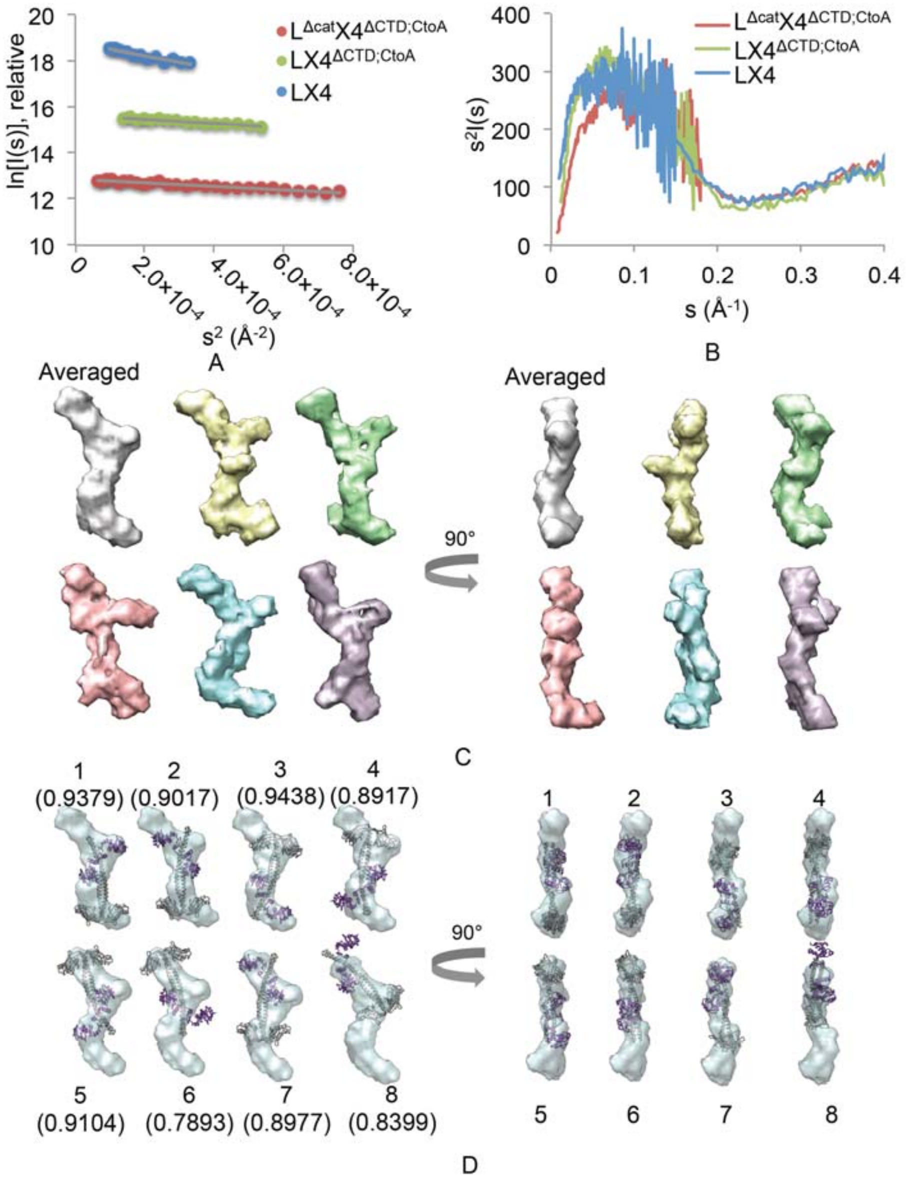

Figure S2, related to Figure 2. Guinier and Kratky plots of LX4, LX4$^{\Delta CTD;CtoA}$ and L$^{\Delta cat}$X4$^{\Delta CTD;CtoA}$. (A) Guinier plots of LX4, LX4$^{\Delta CTD;CtoA}$ and L$^{\Delta cat}$X4$^{\Delta CTD;CtoA}$. The figure shows the Guinier plots of LX4 (blue), LX4$^{\Delta CTD;CtoA}$ (green) and L$^{\Delta cat}$X4$^{\Delta CTD;CtoA}$ (red) in their Guinier regions ($sR_g < 1.3$). The Guinier plots of the latter two constructs were modified after Ochi et al. 2010. (B) Kratky plots of LX4, LX4$^{\Delta CTD;CtoA}$ and L$^{\Delta cat}$X4$^{\Delta CTD;CtoA}$. The figure shows the Kratky plot of the collected SAXS data of LX4 (blue), LX4$^{\Delta CTD;CtoA}$ (green) and L$^{\Delta cat}$X4$^{\Delta CTD;CtoA}$ (red). (C) 3D envelopes of LX4$^{\Delta CTD;CtoA}$. The averaged envelope (grey) (Ochi

et al. 2010) was presented with five single envelopes created using DAMMIN (colored). (D) Fitting of $L^{\Delta cat}X4^{\Delta CTD}$ into the averaged envelope. The figure shows eight-different possible orientations of the structure of $L^{\Delta cat}X4^{\Delta CTD}$ (PDB code: 3II6; Wu et al. 2009) with the envelope. Correlation coefficients of the fittings were calculated using Chimera and are shown in brackets.

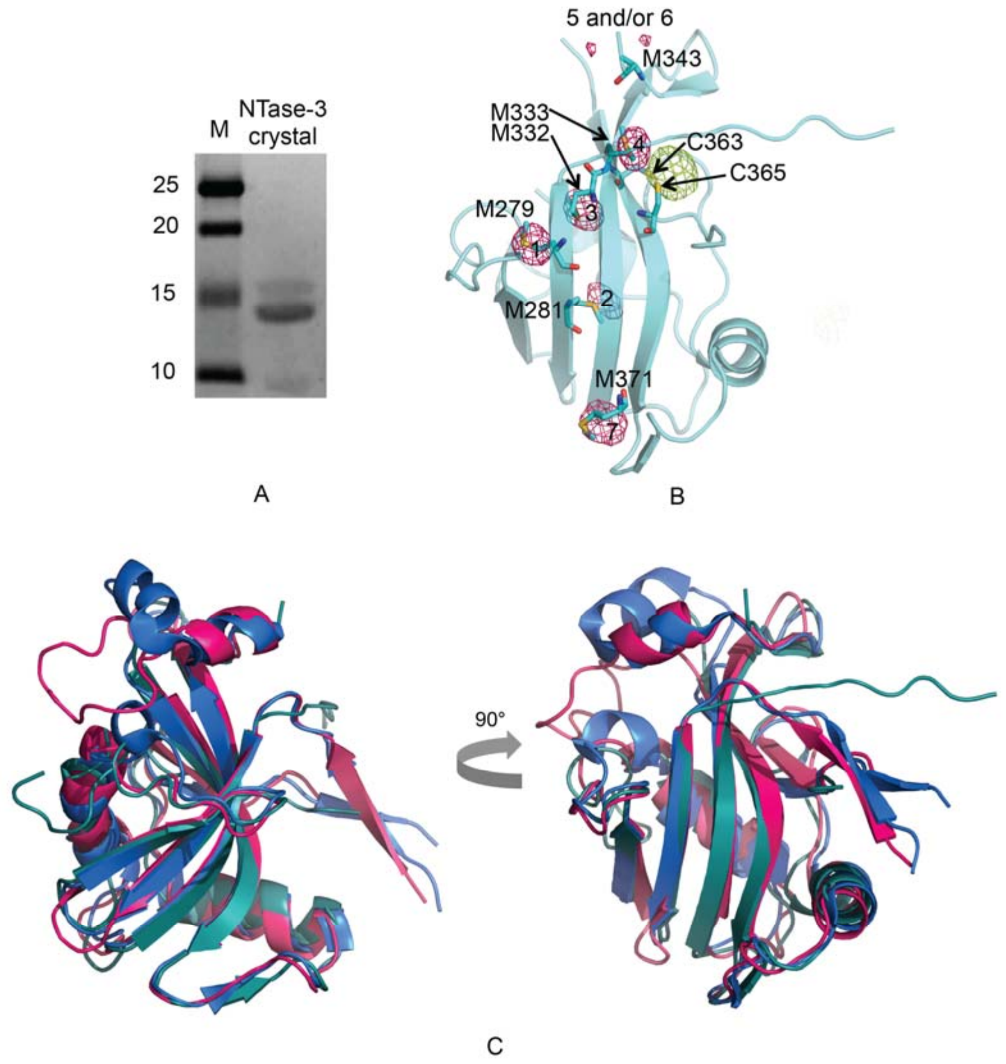

Figure S3, related to Figure 3. Crystallographic structure of NTase-3 of human LigIV. (A) SDS-PAGE of the dissolved SeMet crystals of NTase-3 of human LigIV. (B) Density for of SeMet and Hg from anomalous difference maps calculated from their experimentally obtained phases. Anomalous difference density (contoured at σ=4.0) of SeMet (magenta) and Hg (yellow) are shown with the structure of NTase-3 of human LigIV. (C) Comparison of DNA ligases. Comparison of the structures of NTase-3 of human DNA ligases. They are from the left: human LigI (magenta), LigIII (blue) and LigIV (aqua green). Root mean square differences between LigIV (residue 276-402) and LigI (residue 571-695), and LigIII (residue 424-543) were 1.5 and 1.8 Å respectively. These were calculated using the Dali server (Hasegawa and Holm 2009).

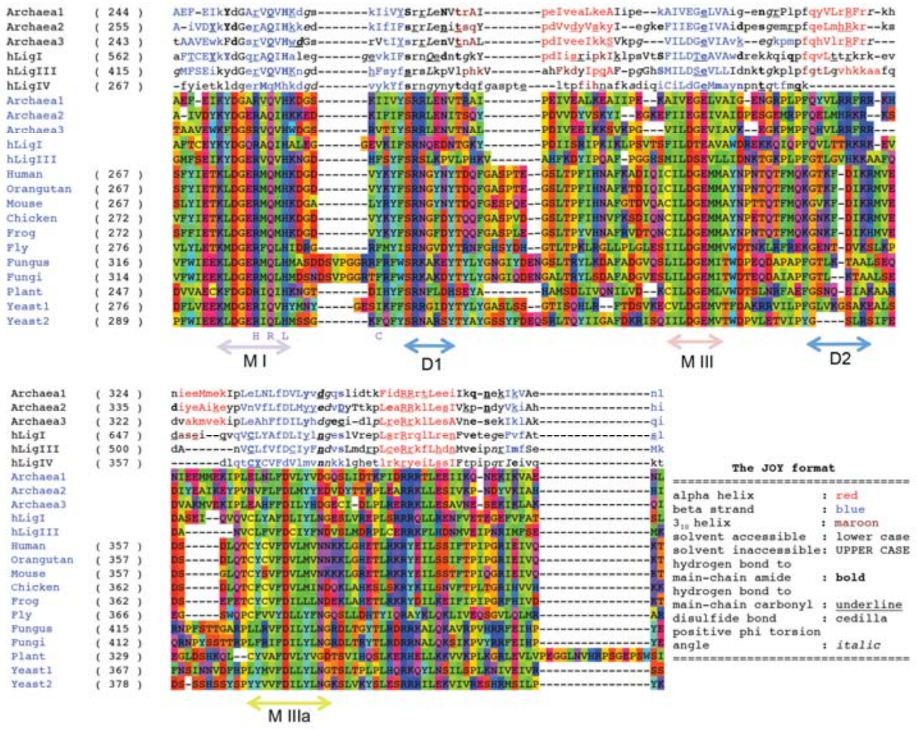

Figure S4, related Figure 5. A structure-based sequence alignment of NTase-3 of ATP-dependent DNA ligases. Sequence alignment of DNA ligase IV and other DNA ligases. Three DNA ligases from archaea (PDB codes: 2HIX; PASCAL et al., 2006, 2CFM; NISHIDA et al., 2006 and 3GDE; KIM et al., 2009) and human LigI (PDB code: 1X9N; Pascal et al., 2004) and LigIII (PDB code: 3L2P; Cotner-Gohara et al., 2010) are aligned with LigIV from various organisms in the JOY format (Mizuguchi et al., 1998). The figure also shows putative DNA-binding regions D1 and D2 using blue arrows. LIG4 syndrome mutations are shown in purple (Chistiakov et al., 2009). The conserved motif I (M I), III (M III) and IIIa (M IIIa) are indicated using purple, pink and yellow arrows respectively (Shuman and Schwer, 1995).

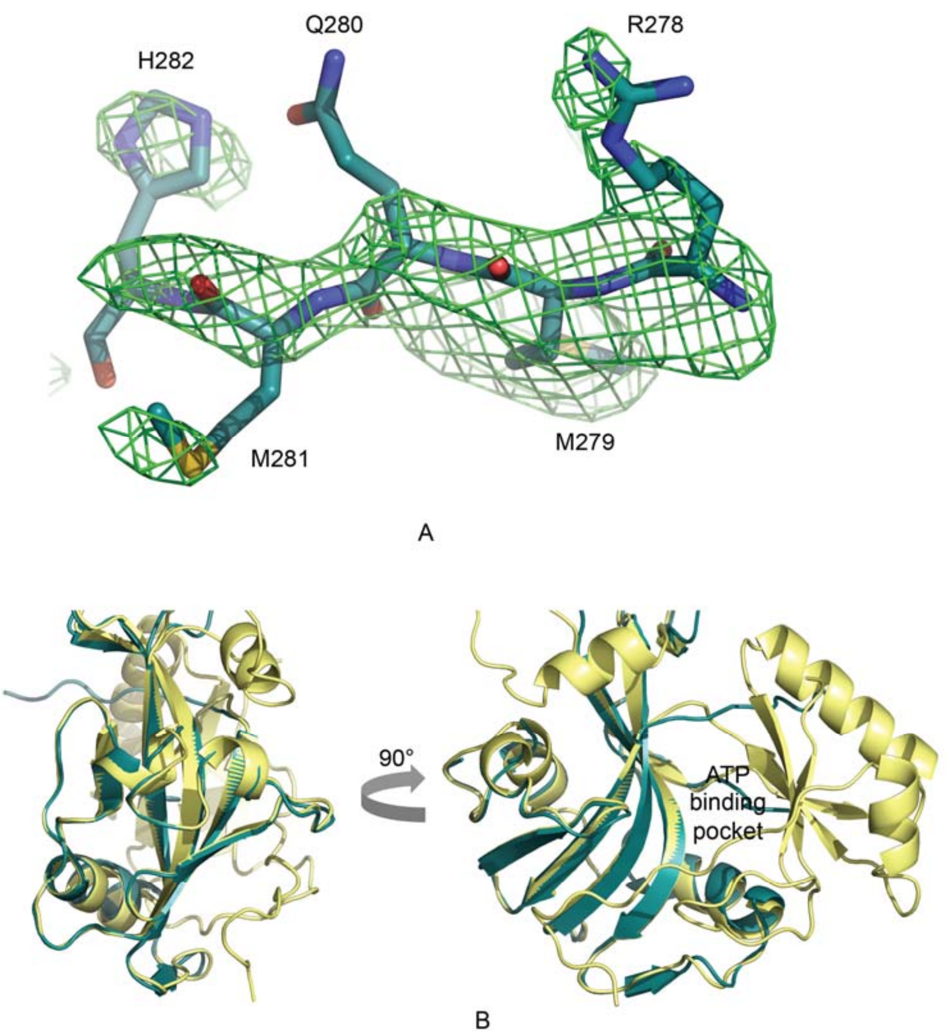

Figure S5, related to Figure 6. Model of NTase of human LigIV and mapping onto the structure of LIG4 syndrome-associated residues. (A) A conserved RXQXH motif (X is a hydrophobic residue) in β1 of NTase-3. The structure of LigIV (cyan) is compared to that of LigI (pink) and LigIII (blue). Omit maps (contoured at σ=2.0) of the residues are shown in a mesh representation (green). (B) Model of NTase of LigIV. The crystallographic structure of NTase-3 of LigIV (green) was superimposed onto the NTase-3 of whole NTase (yellow).

Supplemental Experimental Procedures

Purification of DBD and OBD

Cells that expressed DBD or OBD were resuspended in 5 ml of a lysis buffer (50 mM Tris-HCl pH 8 at 4 °C, 300 mM NaCl, 5 mM DTT, 1x Complete, EDTA-free Protease Inhibitor Cocktail Tablets (Roche)) per gram of cells. Lysis was by sonication using (Misonix). The cell debris was removed by centrifugation at 32,000 g and 4 °C for 30 min. The supernatant was passed through a 0.45 μm membrane filter (Satorius stedim) before loading on the GSTrap FF 10/16 column (GE Healthcare), which was equilibrated with GST buffer A (the lysis buffer without the protease inhibitors). After the sample was loaded onto the column, it was washed with buffer A, and the bound proteins were eluted with GST buffer B (buffer A plus 25 mM reduced L-glutathione). The elution was dialysed against 5 L of dialysis buffer A (30 mM Tris-HCl pH 8.0 at 4 ˚C, 150 mM NaCl, 3 mM DTT, 1 mM EDTA) followed by 5L of dialysis buffer B (50 mM Tris-HCl pH 8.0 at 4 ˚C, 150 mM NaCl, 1 mM β-mercaptoethanol) The dialysed sample was loaded onto a 5 ml HisTrap FF column (GE Healthcare), which was equilibrated with the dialysis buffer. The column was washed with the dialysis buffer followed by Ni buffer A (50 mM Tris-HCl pH 8.0 at 4 ˚C, 300 mM NaCl, 5 mM imidazole for DBD and 26 mM imidazole for OBD) and Ni buffer B (Ni buffer A but 300 mM imidazole). The elution was concentrated to less than 250 µl using Amicon Ultra-15, 10 kDa (Millipore) and loaded onto a Superdex75 10/300 column (GE Healthcare). A single peak was collected and concentrated to 10 mg/ml and snap-frozen with liquid nitrogen for storage at -80 ˚C.

Purification of NTase

Cells that expressed NTase were lysed as described in the purification of DBD, but in a different lysis buffer (50 mM sodium phosphate pH 7.4, 300 mM NaCl, 20 mM imidazole, 10 %(v/v) glycerol, 1x Complete, EDTA-free Protease Inhibitor Cocktail Tablets). The supernatant was passed through 0.45 µm membrane filter and loaded onto a 5 ml HisTrap column, which was

pre-equilibrated with Ni buffer A (50 mM sodium phosphate pH 7.4, 300 mM NaCl, 20 mM imidazole, 10 %(v/v) glycerol), by using a peristaltic pump (Amersham bioscience). After the column was washed with Ni buffer A for 10 CV, weakly bound molecules were removed with Ni buffer B (50 mM sodium phosphate pH 7.4, 300 mM NaCl, 35 mM imidazole, 10 %(v/v) glycerol). The bound molecules were eluted with Ni buffer C (50 mM sodium phosphate pH 7.4, 300 mM NaCl, 300 mM imidazole, 10 %(v/v) glycerol). The elution was dialysed against 5 L of Q buffer A (50 mM Tris-HCl pH 7.5 at 4 °C, 10 mM NaCl, 5 mM DTT) overnight. The dialysed sample was loaded onto a 5 ml HiTrap Q HF column (GE Healthcare), which was pre-equilibrated with Q buffer A. The column was washed with Q buffer A until the absorbance of 280 nm light was stabilised. The bound molecules were eluted with a concentration gradient of Q buffer B (50 mM Tris-HCl pH 7.5 at 4 °C, 1000 mM NaCl, 5 mM DTT). The fractions that contained NTase were collected and concentrated to less than 1 ml. The concentrated sample was centrifuged at 13,000 rpm, 4 °C for 10 min before being injected into a 2 ml loop. The sample was loaded onto a Superdex 75 16/60 column (GE Healthcare), which was pre-equilibrated with 20 mM Tris-HCl pH 7.5 at 4 °C, 300 M NaCl, 5 mM DTT, and a single peak was collected. The collected sample was diluted two-fold with 20 mM Tris-HCl pH 7.5 at 4 °C, 5 mM DTT and concentrated to 10 mg/ml before being snap-frozen with liquid nitrogen and stored at -80 °C. For the purification of the selenium methionine derivative of NTase (SeMetNTase), the same protocol was followed but the DTT concentration was increased to 10mM. Additionally, 10 mM β-mercaptoethanol was included in buffers used in the Ni column step.

Purification of $L^{\Delta cat}X4^{\Delta CTD;CtoA}$ and $L^{\Delta cat}X4$

Cells expressing $L^{\Delta cat}X4^{\Delta CTD;CtoA}$ and $L^{\Delta cat}X4$ were lysed as described in the purification of DBD, but in a different lysis buffer (50 mM Tris-HCl pH 7.5 at 4 °C, 300 (or 1000 mM for $L^{\Delta cat}X4$) KCl, 10 mM imidazole, 1x Complete, EDTA-free Protease Inhibitor Cocktail Tablets (plus 5 mM β-mercaptoethanol for $L^{\Delta cat}X4$)). The cell debris was removed as described in the purification of DBD. The supernatant was passed through 0.45 μm membrane

filter and loaded onto a 5 ml HisTrap column, which was pre-equilibrated with the lysis buffer, using a peristaltic pump. After the column was washed with the lysis buffer for 10 CV, weakly bound molecules were removed with Ni-wash buffer 1 and 2 (50 mM Tris-HCl pH 7.5 at 4 °C, 300 mM KCl, 10 and 20 mM imidazole respectively (plus 5 mM β-mercaptoethanol for $L^{\Delta cat}X4$)). The bound molecules were eluted with the Ni-elute buffer (50 mM Tris-HCl pH 7.5 at 4 °C, 300 mM KCl, 200 mM imidazole (plus 5 mM β-mercaptoethanol for $L^{\Delta cat}X4$)). The elution was dialysed against 5 L of buffer A (50 mM Tris-HCl pH 7.5 at 4 °C, 5 mM KCl, (plus 10 mM DTT for $L^{\Delta cat}X4$)) overnight. The dialysed sample was filtered with a 0.22 μm membrane filter then loaded onto a 6 ml Resource Q column (GE Healthcare), which was pre-equilibrated with buffer A with 10 mM DTT. The column was washed with buffer A until the UV absorbance profile (at 280nm wavelength) became flat. The bound molecules were eluted with a linear concentration gradient of buffer B (50 mM Tris-HCl pH 7.5 at 4 °C, 1000 mM KCl, 0.5 mM (10 mM for $L^{\Delta cat}X4$) DTT). The fractions that contained $L^{\Delta cat}X4$ were collected and concentrated to less than 1 ml. The concentrated sample was centrifuged at 13,000 rpm, 4 °C for 10 min before injection into a 2 ml sample loop. The sample was loaded onto a Superdex 200 16/60 column pre-equilibrated with 20 mM Tris-HCl pH 7.5 at 4 °C, 300 M KCl, 0.5 mM (10 mM for $L^{\Delta cat}X4$) DTT, and a single peak was collected. The collected sample was diluted two-fold with 20 mM Tris-HCl pH 7.5 at 4 °C, 0.5 mM (10 mM for $L^{\Delta cat}X4$) DTT and concentrated to 10 mg/ml before being snap-frozen with liquid nitrogen for storage at -80 °C.

Purification of $LX4^{\Delta CTD;CtoA}$ and $L^{\Delta DBD}X4^{\Delta CTD;CtoA}$

The purification protocols of $LX4^{\Delta CTD;CtoA}$ and $L^{\Delta DBD}X4^{\Delta CTD;CtoA}$ iare based on a reported protocol for the purification of LX4 (Wang et al., 2007). Cells that expressed $LX4^{\Delta CTD;CtoA}$ or $L^{\Delta DBD}X4^{\Delta CTD;CtoA}$ were lysed as described in the purification of DBD, but in a different lysis buffer (50 mM Na Phosphate pH 8.0, 300 mM NaCl, 10 %(v/v) glycerol, 1 mM β-mercaptoethanol, 100 μM PMSF, 100 μM AEBSF, 1x Complete, EDTA-free Protease Inhibitor Cocktail Tablets). The cell debris was removed as described in the purification of

DBD. The supernatant was loaded onto 5 ml HisTrap columns pre-equilibrated with the lysis buffer by using a peristaltic pump. After the columns were washed with the lysis buffer for 10 CV, weakly bound molecules were removed with Ni-wash buffers described in Wang et al., 2007. The bound molecules were eluted with a Ni-elute buffer (50 mM Na phosphate pH 7.0, 300 mM NaCl, 10 %(v/v) glycerol, 1 mM β-mercaptoethanol, 200 mM imidazole). The elution was concentrated to a volume smaller than 5 ml and diluted to 50 ml by Hep buffer A (20 mM Tris-HCl pH 8.0 at 4 °C, 100 mM NaCl, 10 %(v/v) glycerol, 5 mM DTT, 0.1 mM EDTA). The sample was loaded onto 5 ml HiTrap Heparin columns (GE Healthcare) pre-equilibrated with Hep buffer A, after filtrated with 0.22 μm membrane filter. The column was extensively washed with Hep buffer A. The bound molecules were eluted with a linear concentration gradient of Hep buffer B (20 mM Tris-HCl pH 8.0 at 4 °C, 1 M NaCl, 10 %(v/v) glycerol, 5 mM DTT, 0.1 mM EDTA). The fractions containing LmX4 were collected and concentrated to a volume smaller than 5 ml and diluted with HA buffer A (50 mM Na phosphate pH 7.0, 2 %(v/v) glycerol, 10 mM DTT, 0.5 %(v/v) Tween 20) to 50 ml. The sample was loaded onto a 5 ml hydroxyapatite column (Bio-Rad) pre-equilibrated with HA buffer A. After extensively washed with HA buffer A, the bound molecules were washed with HA buffer B (130 mM Na phosphate pH 7.0, 2 %(v/v) glycerol, 10 mM DTT, 0.5 %(v/v) Tween 20). The column was further washed with HA buffer C (100 mM Na phosphate pH 7.0, 2 %(v/v) glycerol, 10 mM DTT), and the bound molecules were eluted with a linear concentration gradient of HA buffer D (230 mM Na phosphate pH 7.0, 2 %(v/v) glycerol, 10 mM DTT). When hydrophobic interaction columns were used instead of the hydroxyapatite column, the collected fractions from the heparin column were diluted three-fold with Phe buffer A (50 mM Na phosphate pH 7.0, 5 %(v/v) glycerol, 5 mM DTT, 1 M ammonium sulfate) and were loaded onto a HiTrap Phenyl HP column (GE healthcare). The bound proteins were eluted with a linear concentration gradient of Phe buffer B (120 mM Na phosphate pH 7.0, 5 %(v/v) glycerol, 5 mM DTT). A single peak was concentrated to a volume smaller than 1 ml and centrifuged at 13,000 rpm, 4 °C for 10 min before injected into a 2 ml loop. The sample was loaded onto a Superdex 200 16/60 column (GE Healthcare),

which was pre-equilibrated with a S200 buffer (20 mM Tris-HCl pH 8.0 at 4 °C, 200 M NaCl, 5 %(v/v) glycerol, 5 mM DTT). A single peak was concentrated to 10 mg/ml before snap-frozen with liquid nitrogen and stored at -80 °C.

Purification of LX4

LX4 was firstly purified using Ni columns as described in the purification of $LX4^{\Delta CTD;CtoA}$. The elution was concentrated to a volume smaller than 5 ml and diluted to 50 ml by Q buffer A0 (50 mM Tris-HCl pH 8.0 at 4 °C, 50 mM NaCl, 10 %(v/v) glycerol, 5 mM DTT, 1 mM EDTA). The sample was loaded onto a 6 ml Resource Q column pre-equilibrated with Q buffer A (50 mM Tris-HCl pH 8.0 at 4 °C, 100 mM NaCl, 10 %(v/v) glycerol, 5 mM DTT, 1 mM EDTA) after being filtrated with 0.22 μm membrane filter. The column was extensively washed with Q buffer A. The bound molecules were eluted with a linear concentration gradient of Q buffer B (50 mM Tris-HCl pH 8.0 at 4 °C, 1 M NaCl, 10 %(v/v) glycerol, 5 mM DTT, 1 mM EDTA). The eluted fractions were diluted three-fold with Hep buffer A0 (50 mM Tris-HCl pH 8.0 at 4 °C, 10 %(v/v) glycerol, 5 mM DTT, 1 mM EDTA) and loaded onto a 5 ml HiTrap Heparin column by using a peristaltic pump. After the column had been washed with Q buffer A, the bound molecules were eluted with Q buffer B. The elution was concentrated to a volume smaller than 1 ml and centrifuged at 13,000 rpm, 4 °C for 10 min before injected into a 2 ml loop. The sample was loaded onto a Superdex 200 16/60 column, which was pre-equilibrated with a S200 buffer (20 mM Tris-HCl pH 8.0 at 4 °C, 200 M NaCl, 5 %(v/v) glycerol, 5 mM DTT). A single peak was collected and concentrated to a volume smaller than 5 ml and diluted with HA buffer A (50 mM Na phosphate pH 7.0, 2 %(v/v) glycerol, 10 mM DTT, 0.5 %(v/v) Tween 20) to 50 ml. The sample was loaded onto a 5 ml hydroxyapatite column, which was pre-equilibrated with HA buffer A. After extensively washed with HA buffer A, the bound molecules were washed with HA buffer B (130 mM Na phosphate pH 7.0, 2 %(v/v) glycerol, 10 mM DTT, 0.5 %(v/v) Tween 20). The column was further washed with HA buffer C (100 mM Na phosphate pH 7.0, 2 %(v/v) glycerol, 10 mM DTT), and the bound molecules were eluted with a linear concentration gradient of HA buffer

D (230 mM Na phosphate pH 7.0, 2 %(v/v) glycerol, 10 mM DTT). A single peak was collected, and its buffer was exchanged to the S200 buffer by using a PD-10 column (GE Healthcare). The sample was concentrated to 100 μM before being snap-frozen with liquid nitrogen and stored at -80 °C.

Small angle X-ray scattering

Two sample-to-detector distances (1 and 4.25 m) were used to collect the scattering intensity in the range of the momentum transfer interval 0.012 $Å^{-1} < s$ $(=4\pi\sin 2\theta/\lambda) < 0.81$ $Å^{-1}$, where θ is the scattering angle and λ is the wavelength of the incident beam (1.54 Å at station 2.1). SAXS data were collected at different protein concentrations in order to assess possible macromolecular aggregation. Thus LX4 was measured at concentrations between 1 and 5.6 mg/ml in 20 mM Tris-HCl pH 8.0 at 4 °C, 200 mM NaCl, 5 mM DTT, 5 %(w/v) glycerol; for $LX4^{\Delta CTD;CtoA}$, which does not have X4CTD (residue 214-334) and in which all cysteines (C93, C128, C130 and C165) are mutated to alanines, concentrations of 1 and 7.5 mg/ml in the same buffer were used; and for $L^{\Delta cat}X4^{\Delta CTD;CtoA}$, which is $LX4^{\Delta CTD;CtoA}$ but does not have the catalytic region of LigIV (residue 1-653), concentrations between 1 and 10 mg/ml in 20 mM Tris-HCl pH 7.5 at 4 °C, 150 mM KCl, 5 mM DTT were used. After centrifuging the samples at 13,000 rpm 4 °C for 10 min, about 100 μl of each sample was loaded in the temperature controlled sample cell and exposed to the beam in frames of 60 sec for up to 45 min total measuring time. During experiments the temperature of the cell was kept at 4 ºC. Subtraction of the buffer scattering and data reduction were performed using beamline-specific software at the Daresbury SRS. Ten individual models created using DAMMIN (Svergun, 1999) were superimposed and averaged using SUPCOMB (Kozin and Svergun, 2001) and DAMAVER (Volkov and Svergun, 2003). For rigid-body modeling of $LX4^{\Delta CTD;CtoA}$, 10 Å distance restraints were forced between I238 and S239, L75 and Y296, N216 and F246, V217 and F246, and E453 and D460 that do not change the relative distances greatly, as indicated by comparison of open, closed and DNA-bound forms of the catalytic region of LigIV. The conformational models were created by superimposing modeled DBD, NTase and OBD of

LigIV on the crystal structure of an archaeal DNA ligase (PDB code: 2HIX for open or 2CFM for closed) or human LigI (PDB code: 1X9N). Similar results were obtained without the distance constraints described (data not shown). Ten individual models were generated. The discrepancy χ^2 is defined as (Svergun et al., 1995)

$$\chi^2 = \frac{1}{N-1}\sum_i \left[\frac{I_{exp}(s_i) - cI_{calc}(s_i)}{\sigma(s_i)}\right]^2$$

where N is the number of measured intensity points, $I_{exp}(s_i)$ is the observed scattering intensity, c is a scaling factor, $I_{calc}(s_i)$ is the calculated scattering intensity, $\sigma(s_i)$ is the error of the observed scattering intensity. For both DAMMIN and BUNCH, the SAXS data up to 0.4 Å^{-1} were used. The UCSF Chimera package (Pettersen et al., 2004) was used to visualize the model envelopes, to fit crystallographic models into the envelopes and to calculate correlation coefficients of the fittings.

Structural solution and model building

The initial set of phases, the figure of merit (FOM) of which was 0.38, was obtained from the SeMet diffraction data using the SAD method, which identified five out of seven selenomethionines: M279, M281, M332, M333 and M371. Other sets of phases were determined by the SIRAS method using combinations of 1) Os and Hg data sets (Os/Hg phases) and 2) native-2 and Hg data sets (native-2/Hg phases), FOMs of which were 0.52 and 0.34 respectively. Os and native-2 were considered as native data. Multi-crystal averaging of native-1, the SeMet and the Os/Hg data was carried out using DMMULTI (Cowtan, 1994) in order to calculate phases for native-1 at the maximum resolution of 3.5 Å.

The model of NTase-3 was built using Coot (Emsley et al., 2010). The initial model of NTase-3 was built using the electron density map calculated from the phases obtained from the SeMet SAD data. Refmac 5.5 (Murshudov et al., 2011) and Buster/TNT (Blanc et al., 2004) were used for the initial refinement of the model. The phenix.refine and

phenix.autobuilt modules in PHENIX suite (Adams et al., 2010) and Refmac 5.5 were used for further refinement. The remaining part of NTase-3 was built using the electron density maps calculated for the phases obtained from the Os/Hg data using SIRAS and from the multi-crystal averaging described earlier. At a later stage of the model building, the map calculated from native-2/Hg phases was used to build residues between 373 and 377. The refinement of the new model was carried out against native-1 data up to 3.5 Å resolution using phenix.refine module of PHENIX with secondary structure and Ramachandran restraints. Omit maps were calculated at 2.9 Å resolution by removing sequential five residues from the model in order to fix coordinate errors after the refinement of the model against the 2.9 Å native data using phenix.refine. After a few repeats of rebuilding using Coot and refinement using the jelly-body refinement, the sigma value of which was 0.05, in Refmac 5.6 (Murshudov et al., 2011), the whole structure was re-built using the auto.built module in PHENIX. The best re-built model was refined using torsion-angle simulated annealing at 2500 K. The re-building and refinement was then repeated several times. The anomalous different map using the SeMet data was calculated again using the refined model and showed two additional weak peaks corresponding Se atoms in M343 and M354. This allowed us to build a loop (residue 336-344) more accurately than previous models; however, the electron density of the side chain of M343 and that around M354 were not observed in map calculated from the 2.9 Å native-1 data. The statistics of the final-refined model of NTase-3 is presented in Table 1. Omit maps were calculated using PHENIX by removing residue 278-282 followed by three cycles of simulated annealing at 5000 K.

Supplemental Reference

1. Adams, P. D. Afonine, P. V., Bunkóczi, G., Chen, V. B., Davis, I. W., Echols, N., Headd, J. J., Hung, L. W., Kapral, G. J., Grosse Kunstleve, R. W., et al. (2010). PHENIX: a comprehensive Python-based system for macromolecular structure solution. Acta Cryst. D 66, 213-221

2. Cowtan, K. (1994). “dm”: An Automated Procedure for Phase Improvement by Density Modification. Joint CCP4 and ESF-EACBM Newsletter on Protein Crystallography 31, 34-38.

3. Hasagawa, H. and Holm, L. (2009). Advances and pitfalls of protein structural alignment. Curr. Opinion Struct. Biol. 19, 341-348

4. Kozin, M. B., and Svergun, D. I. (2001). Automated matching of high- and low-resolution structural models. J. Appl. Cryst. 34, 33-41.

5. Murshudov, G. N., Skubák, P., Lebedev, A. A., Pannu, N. S., Steiner, R. A., Nicholls, R. A., Winn, M. D., Long, F., and Vagin, A. A. (2011). REFMAC5 for the refinement of macromolecular crystal structures. Acta Cryst. D 67, 355-367.

6. Ochi, T., Sibanda, L. B., Wu, Q., Chirgadze, D. Y., Bolanos-Garcia, V. M., and Blundell, T. L. (2010). Structural Biology of DNA Repair: Spatial Organisation of the Multicomponent Complexes of Nonhomologous End Joining. J. Nucleic Acids 2010, 1-19.

7. Pettersen, E. F., Goddard, T. D., Huang, C. C., Couch, G. S., Greenblatt, D. M., Meng, E. C., and Ferrin, T. E. (2004). UCSF chimera-a visualization system for exploratory research and analysis. J. Comput. Chem. 25, 1605-1612.

8. Svergun, D., Barberato, C., and Koch, M. H. J. (1995). CRYSOL - a Program to Evaluate X-ray Solution Scattering of Biological Macromolecules from Atomic Coordinates. J. Appl. Cryst. 28, 768-773.

9. Svergun, D (1999). Restoring Low Resolution Structure of Biological Macromolecules from Solution Scattering Using Simulated Annealing. Biophys. J. 76, 2879-2886.

10. Volkov, V. V., & Svergun, D. I. (2003). Uniqueness of *ab initio* shape determination in small-angle scattering. J. Appl. Cryst. 36, 860-864.

11. Wang, Y., Lamarche, B. J., and Tsai, M. D. (2007). Human DNA ligase IV and the ligase IV/XRCC4 complex: analysis of nick ligation fidelity. Biochemistry 46, 4962-4976.
